# Aberrant expression of CD133 protein correlates with Ki-67 expression and is a prognostic marker in gastric adenocarcinoma

**DOI:** 10.1186/1471-2407-10-218

**Published:** 2010-05-20

**Authors:** Po Zhao, Yazhuo Li, Yali Lu

**Affiliations:** 1 Department of Pathology, Chinese People's Liberation Army General Hospital, Beijing 100853, China

## Abstract

**Background:**

The relationships between the expression of CD133, Ki-67 and prognosis in gastric adenocarcinoma are unknown and needs exploring.

**Methods:**

The samples of gastric adenocarcinoma from 336 Chinese patients with follow-up were analyzed for CD133 and Ki-67 protein expressions by immunohistochemical method.

**Results:**

CD133 was expressed in up to 57.4% (193/336) of this group of gastric carcinoma. The expression of CD133 was significantly higher in carcinoma than in normal (*P *= 0.0001) and dysplastic mucosas (*P *= 0.004). CD133 was positive corresponded with the tumour size, grade, infiltrative depth and clinical stage (all *P *< 0.05). The overall mean survival time of the patients with CD133 positive expression was shorter than that of patients with negative expression (*P *= 0.0001). The expression of CD133 has a positive correlation with that of Ki-67 (r = 0.188, *P *= 0.001) in gastric adenocarcinoma. CD133 was an independent prognostic indicator. (*P *= 0.0001).

**Conclusions:**

It is suggested that CD133 may play an important role in the evolution of gastric adenocarcinoma and should be considered as a potential marker for the prognosis.

## Background

Gastric cancer is one of the most aggressive malignancies, with an extremely poor prognosis, and is the second leading cause of cancer death worldwide [1]. The prevalence of gastric cancer in China is amongst the highest in the world, along with Japan and Korea. Lowest rates are associated with the more developed regions such as Northern Europe and North America. By the time the patient is clinically diagnosed, the gastric cancer has often grown beyond the limits of curative resection. This reality has raised therapeutic problems, and new early diagnostic tools and therapeutic techniques concerning this disease are urgently needed. CD133 is a pentaspan transmembrane glycoprotein, with a molecular weight of 120 kDa [2]. Although it was initially considered to be one marker of hematopoietic stem cells, CD133 mRNA transcript could also be found in normal non-lymphoid hematopoietic tissue [2]. CD133 is overexpressed in various solid tumours [3-7], including colon cancer and glioblastoma [8,9]. O'Brien et al.[9] performed purification of CD133-positive cells in colon carcinoma by flow cytometry and transplanted them into mice, leading to the formation of tumours in renal subcapsule, suggesting that CD133 may be a cancer stem cell marker for colon carcinoma. Recently, Smith and colleagues have shown that a moderate to high percentage of gastric cancer tumor samples have CD133 expression with moderate to strong membranous and apical expression. No significant association with tumor stage or grade was identified but this conclusion was based on a limited number of cases studied.

They identified CD133 as a potential therapeutic target for antibody-drug conjugates in gastric cancer [10], raising the possibility of molecular targeting therapy in this most aggressive malignancy. CD133 expression as a prognostic marker has been found in colorectal cancer [11-18] and brain tumours [19-21] but it is still unclear if CD133 can be used as a prognostic marker in pancreatic cancer [22], ovarian cancer [23], hepatocellular cancer [24,25] and non-small cell lung carcinoma [26]. Whether CD133 is a prognostic marker in gastric cancer is still unclear since to date no extensive study has been performed correlating CD133 expression with clinicopathological features and prognosis. Ki-67 is a well-recognized nuclear antigen-specific marker associated with cellular proliferation, and primarily used to judge the activity of proliferation. The relationship between CD133 and Ki-67 in gastric cancer has not yet been investigated. We performed an immunohistochemical study to investigate the possible role of the expression of CD133 in clinicopathology and prognosis, as well as the relationship between CD133 and Ki-67 in 336 cases of gastric adenocarcinoma. It is hoped that the study will give information on the pathogenesis of this disease.

## Methods

### Biopsy Specimens

Paraffin embedded sections of 336 gastric adenocarcinomas with 58 adjacent dysplastic mucosas and 60 distal normal gastric tissues were obtained from the Department of Pathology, Chinese People's Liberation Army (PLA) General Hospital (Beijing, China). Ethical approval for this study was not required by our institution as the experiments carried out did not relate to patient's privacy, impairment or treatment. The age of the patients ranged from 18-85 years, with an average of 58.1 years. Two hundred seventy-four were men and 62 were women. Seventeen were at grade 1, 65 at grade 2 and 254 at grade 3, according to histological grading. Sixty-six were at stage I, 77 at stage II, 147 at stage III and 46 at stage IV, according to clinical staging of TNM, respectively. Lymphatic metastasis in regional nodes at operation was confirmed in 225 cancers of this study.

### Immunohistochemistry

All samples were fixed in 10% buffered formalin and embedded in paraffin. Sections were cut 4 μM thick from wax blocks, mounted on to APES-coated glass slides. Slides were deparaffinized in xylene twice for 10 minutes, rehydrated through graded ethanols to distilled water before incubation for 15 minutes with 3% hydrogen peroxidase-methanol to inhibit endogenous peroxidase activity, and heated in 0.01 M citrate buffer (pH 6.0) in a microwave oven for 5 minutes at 100°C; after boiling for antigen retrieval. Then the slides were taken out of microwave oven and cooled to room temperature for 30 minutes. After incubating for 15 minutes in a blocking solution containing 10% normal goat serum in PBS, sections were incubated at 4°C overnight in a humidified chamber with rabbit polyclonal antibody to human CD133 (Abcam Inc., Cambridge, MA, USA) diluted 1:100 in blocking solution and mouse mononal antibody to human Ki-67 (Zymed Laboratories Inc., South San Francisco, CA) diluted 1:100 in blocking solution. The sections were rinsed in PBS and incubated for 20 minutes with Polyperoxidase-anti-mouse/rabbit IgG (Zymed). After washing in PBS, 3,3'-Diaminobenzidine was used as the chromogen. Slides were counterstained for 3 minutes with hematoxylin solution. Brain medulloblastoma tissue was used as a positive control, whereas the primary antibody was replaced by 0.01 M PBS as a negative control.

### Evaluation of score

In scoring expression of CD133 and Ki-67 protein, both the extent and intensity of immunopositivity were considered, according to Hao *et al *[27]. The intensity of positivity was scored as follows: 0, negative; 1, weak; 2, moderate; 3, strong. The extent of positivity was scored according to the percentage of cells showing positive staining: 0, < 5%; 1, > 5-25%; 2, > 25-50%; 3, > 50-75%; 4, > 75% of the cells in the respective lesions. The final score was determined by multiplying the intensity of positivity and the extent of positivity scores, yielding a range from 0 to 12. The expression for CD133 and Ki-67 was considered positive when the scores were ≥5.

### Statistical Analysis

Fisher's exact test (two sided), Pearson Chi's square test for trends in proportions, Spearman's correlation coefficient test, and Kaplan-Meier's method with log rank test or Cox Regression method for univariate or multivariate overall survival analysis were used to assess the associations between expression of CD133 or Ki-67 and clinicopathological indices by SPSS 15.0 for Windows (Chicago, IL). A * P *< 0.05 was considered statistically significant.

## Results

### Expression of CD133 in gastric adenocarcinoma

**Figure 1:**
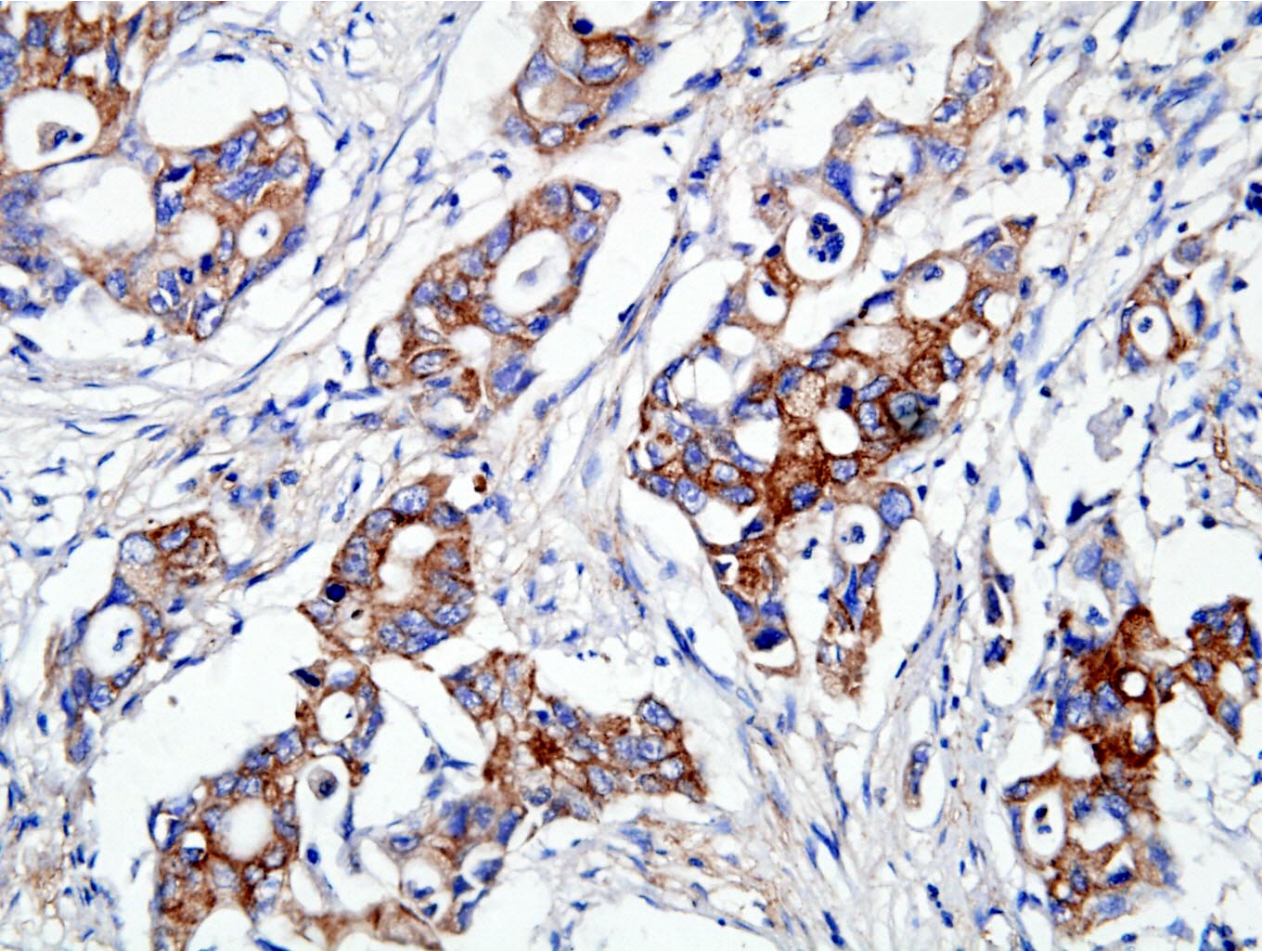
**Expression of CD133 protein in gastric adenocarcinoma**. CD133 expressed positive in the membrane and cytoplasm of cancer cells in poorly-differentiated adenocarcinoma (grade 3). (CD133 ×400).

**Figure 2:**
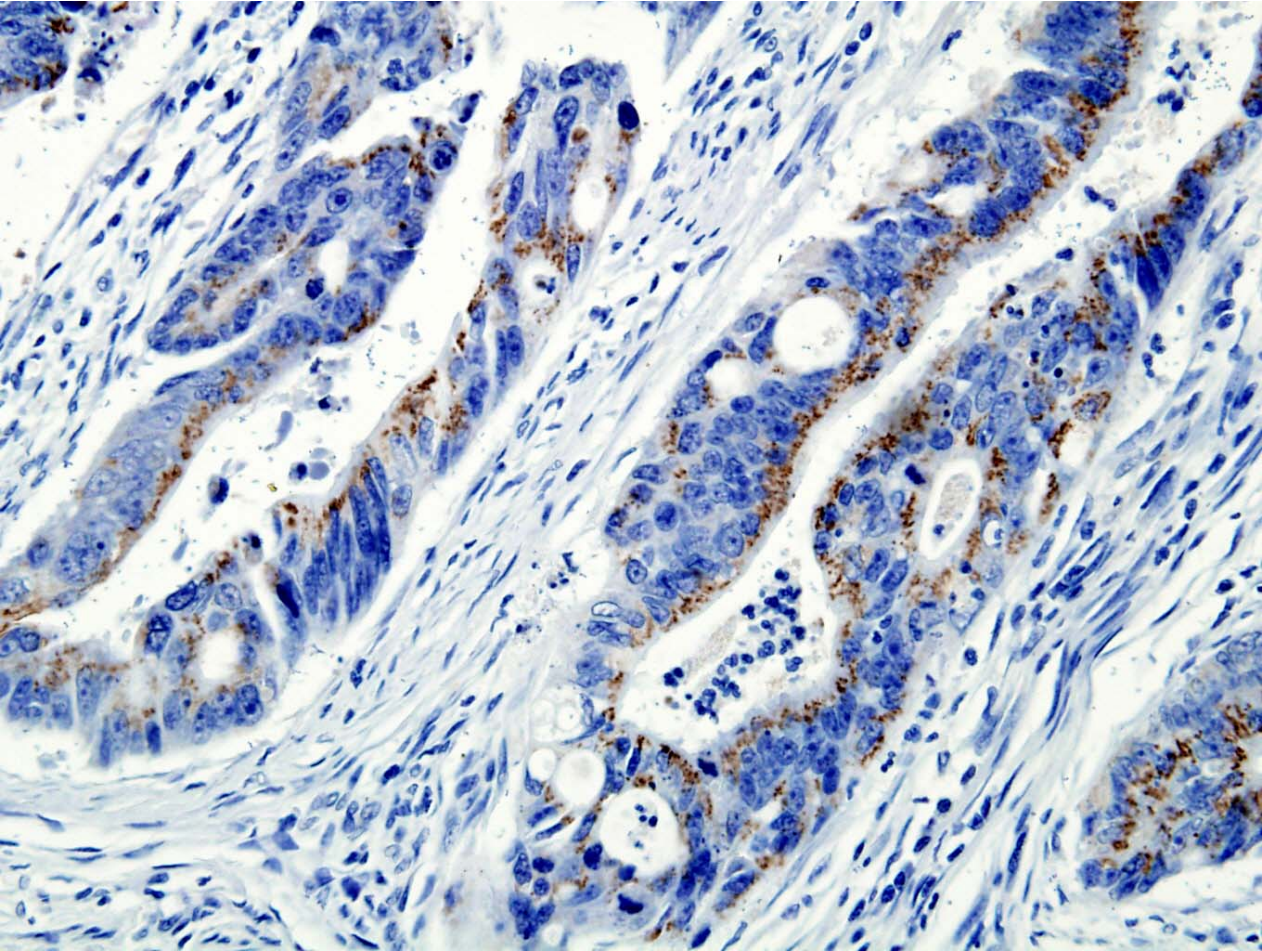
**Expression of CD133 protein in gastric adenocarcinoma**. CD133 predominantly localized in the membrane and cytoplasm around the lumen of cancerous glands in well-differentiated adenoocarcinoma (grade 1). (CD133 ×400).

CD133 protein was expressed positively in 57.4% (193 out of 336) of gastric adenocarcinoma, 36.2% (21 out of 58) of adjacent dysplastic epithelia and 28.3% (17 out of 60) of distal normal mucosa. The localization of CD133 protein was primarily in the membrane and cytoplasm of cancer cells (Figure 1). In well-differentiated adenocarcinoma, CD133 protein was found predominantly around the lumen of cancerous gland (Figure 2). A very significant difference was found in the expression of CD133 protein between gastric adenocarcinoma and normal mucosa or adjacent dysplastic tissues (*P *= 0.0001; *P *= 0.004). There was no difference in CD133 expression between normal mucosa and adjacent dysplastic mucosa (*P *= 0.432).

### The association between expression of CD133 and clinicopathological features

**Figure 3:**
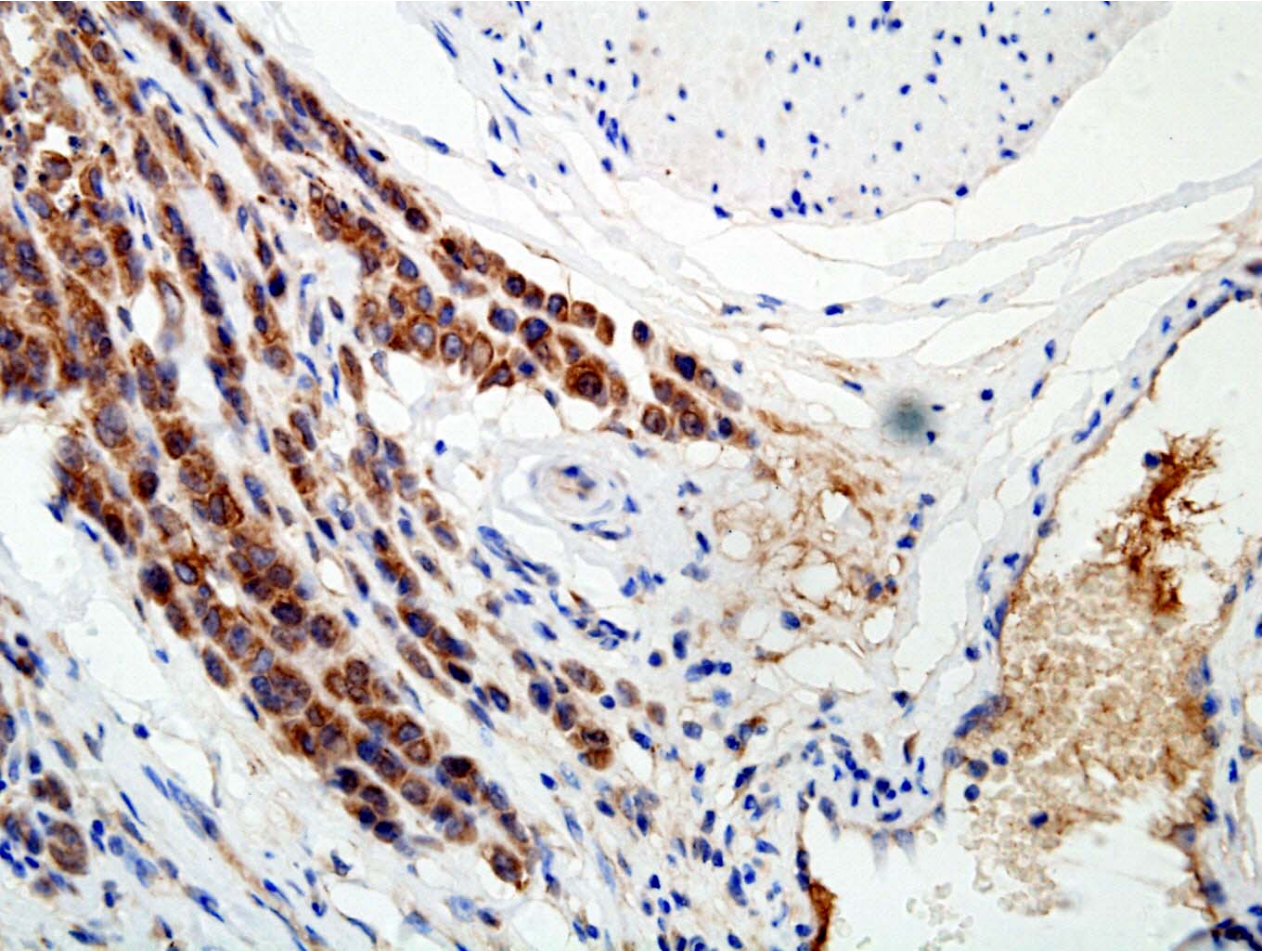
CD133 overexpressed in the membrane and cytoplasm of cancer cells located in invasive depth (CD133 ×400).

**Figure 4:**
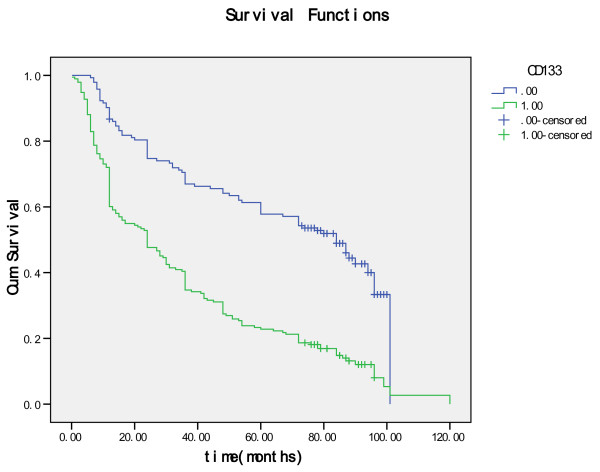
**Kaplan-Meier survival analysis by CD133 status (n = 336)**. The y-axis represents the percentage of patients; the x-axis, their survival in months. The green line represents CD133-positive patients with a trend of worse survival than the blue line representing CD133-negative gastric carcinoma patients (Log rank = 53.80; *P *= 0.0001). Mean survival times were 36.3 months for the CD133- positive group and 66.0 months for the CD133-negative group.

The gastric adenocarcinoma with positive expression of CD133 was 41.2% (7 out of 17) at grade 1, 46.2% (30 out of 65) at grade 2 and 61.4% (156 out of 254) at grade 3, respectively, thus the association between expression of CD133 and histological grade was statistically significant (*P *= 0.032) (Table 1). With respect to the TNM tumor staging adenocarcinoma with positive expression of CD133 was 28.8% (19 out of 66) at stage I, 49.4% (38 out of 77) at stage II, 66.7% (98 out of 147) at stage III and 82.6% (38 out of 46) at stage IV, respectively. Statistically, the positive expression of CD133 correlated with the more advanced clinical stage (*P *= 0.0001). Expression of CD133 was also found to positively correlate with the tumour size (*P *= 0.0001), invasive depth (Figure 3; *P *= 0.0001) and metastasis in the regional lymph nodes (*P *= 0.0001), but not with the age of the patients (*P *= 0.434) (Table 1). Follow-up data showed that a significantly decreasing trend in 5-year survival was observed in patients with the expression of CD133 (*P *= 0.0001) (Table 1). There was also a significant difference in the overall mean survival time between the carcinomas with expression of CD133 (36.4 months) and those without (66.0 months) (Log rank = 53.80; *P *= 0.0001) (Figure 4). CD133 was an independent prognostic factor by multivariate analysis (*P *= 0.0001).

**Table 1:** Relationship between CD133 or Ki-67 and clinicopathological features

Clinicopathological features	CD133		*Probability*	Ki-67		*Probability*
				
	+	-		+	-	
Ages						
n≤40	16	18	0.434	20	14	0.291
40<n≥65	116	82		109	89	
>65	61	43		67	37	
Size						
d<4 cm	30	47	0.0001	32	45	0.001
4 cm≤d<8 cm	117	84		121	80	
d≥8 cm	46	12		43	15	
Grade						
1	7	10	0.032	6	11	0.007
2	30	35		30	35	
3	156	98		160	94	
Invasive depth						
submucosa	4	19	0.0001	6	17	0.001
subserosa	47	56		52	51	
visceral peritoneum	122	65		118	69	
adjacent structures	20	3		20	3	
Lymph nodes						
N0	43	68	0.0001	44	67	0.0001
N1	78	50		77	51	
N2	52	20		52	20	
N3	20	5		23	2	
Stage						
I	19	47	0.0001	20	46	0.0001
II	38	39		40	37	
III	98	49		96	51	
IV	38	8		40	6	
Five-year survival						
Alive	44	82	0.0001	46	60	0.0001
Dead	149	61		150	60	

### The association between CD133 and Ki-67

**Figure 5:**
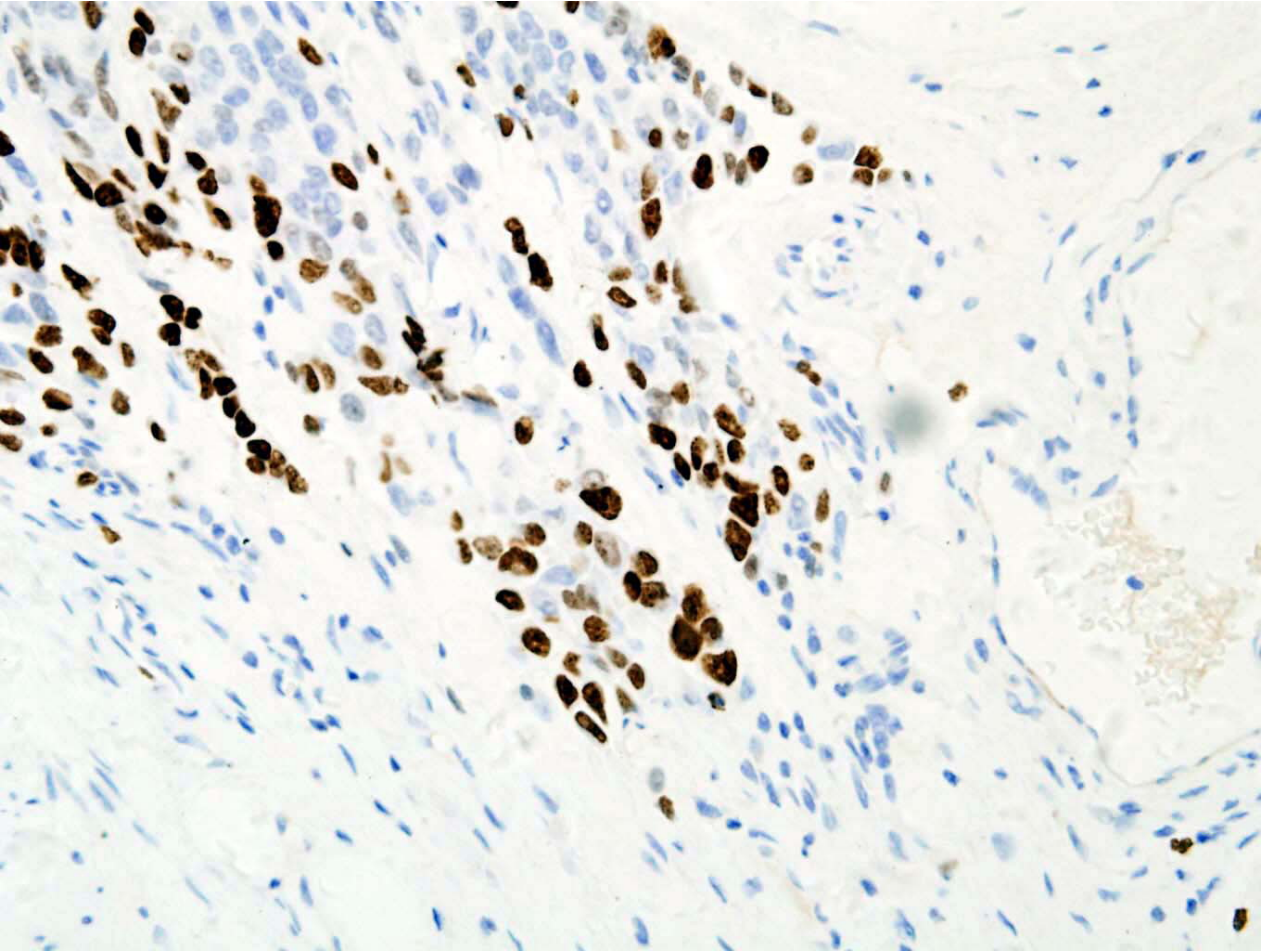
**Ki-67 expressed in the nucleus of cancer cells located in invasive depth (Ki-67 ×400)**.

Ki-67 was positive in 58.3% (196 out of 336) cases of gastric adenocarcinoma and associated with clinicopathological features such as tumour size, invasive depth, metastasis in regional lymph nodes, histological grade, clinical stage and prognosis (Figure 5; Table 1). The positive correlation between CD133 and Ki-67 was found (r = 0.188, *P *= 0.001).

## Discussion

Cancer stem cells, also known as tumour initiating cells, refer to a small side population of tumour cells with unlimited self-renewal activity and potentially promote the formation of tumour, according to the cancer stem cell (CSC) hypothesis that has emerged recently [28-32]. There is currently heightened interest in the utility of using CD133 as a marker to identify the tumor stem cell population for a variety of malignancies [3-5,8] Recently, CD133 was found to be highly expressed in more or equal to 50% of gastric, pancreatic and intrahepatic cholangiocarcinomas [10]. Quantitative flow cytometric analysis showed that a panel of established gastric cancer cell lines expressed CD133 at levels higher than normal epithelial cells. However, it has also been found that CD133 expression is not restricted to stem cells, and both CD133+ and CD133- metastatic colon cancer cells initiate tumors [33,34]. A murine anti-human CD133 antibody conjugated to a potent cytotoxic drug, monomethyl auristatin F (MMAF), effectively inhibited the growth and induced apoptosis of KATO III gastric cancer cells in vitro [10]. Therefore, anti-CD133 antibody-drug conjugates may warrant further evaluation as a molecular therapeutic strategy to eradicate CD133-positive tumour cells in gastric cancer. CD133 expression as a prognostic marker has been found in colorectal cancer [11-18] and brain tumours [19-21], although it is still controversial for ovarian cancer and non-small cell lung cancer. There are no reports on the prognostic significance of CD133 expression or correlation with Ki-67 in gastric cancer. In this study the positive expression of CD133 is found in 57.4% and is closely related to the worst prognosis of gastric adenocarcinoma. Our results indicate that the expression of CD133 may be involved in tumor size, invasive depth and histological grade of gastric tumor, which are in agreement with previous reports on colorectal cancer [16,17]. In this research, the expression of CD133 positively correlates with the shorter survival time, suggesting CD133-positive cells may contain more cancer stem-like cells. Thus residual cancer stem-like cells after routine therapy may lead to tumour recurrence and metastasis through the self-renewal and multilineage differentiation with agreement of cancer stem cell hypothesis [35,36]. It implies that CD133 may be one of the specific molecular markers in prognosis of gastric adenocarcinoma. The results of our study, that Ki-67 expression corresponds with tumor size, degree of differentiation, depth of invasion, lymphatic metastasis, TNM staging and prognosis, are associated with a previous report [37]. This research also shows that CD133 positively correlates with Ki-67, implying higher putative proliferative activity in CD133-positive cancer cells, which corresponds with the results from Ieta, et al. [35]. Taken together, CD133 is highly expressed in the cancer cells of gastric adenocarcinoma. The combined detection of CD133 and Ki-67 expression, to some extent, can reflect the biological behavior of gastric cancer cells, thus guiding the choice of chemotherapy and molecular targeting therapy.

## Conclusions

It is suggested that CD133 may play an important role in the evolution of gastric adenocarcinoma and it can be considered as a potential marker for the prognosis in patients with gastric adenocarcinoma.

## Competing interests

The authors declare that they have no competing interests.

## Authors' contributions

ZP carried out the design, analysis of pathology and drafted the manuscript. LYZ carried out sample collections and coordination. LYL performed the immunohistochemical staining. All authors read and approved the manuscript.

## Pre-publication history

The pre-publication history for this paper can be accessed here:

http://www.biomedcentral.com/1471-2407/10/218/prepub

## References

[B1] Parkin DM Bray F Ferlay J Pisani P Global cancer statistics. 2002 CA Cancer J Clin 2005 55 74 108 10.3322/canjclin.55.2.74 15761078

[B2] Miraglia S Godfrey W Yin AH Atkins K Warnke R Holden JT Bray RA Waller EK Buck DW A novel five-transmembrane hematopoietic stem cell antigen: isolation, characterization, and molecular cloning Blood 1997 90 5013 5021 9389721

[B3] Hilbe W Dirnhofer S Oberwasserlechner F Schmid T Gunsilius E Hilbe G Wöll E Kähler CM CD133 positive endothelial progenitor cells contribute to the tumour vasculature in non-small cell lung cancer J Clin Pathol 2004 57 965 969 10.1136/jcp.2004.016444 15333659 PMC1770433

[B4] Singh SK Hawkins C Clarke ID Squire JA Bayani J Hide T Henkelman RM Cusimano MD Dirks PB Identification of human brain tumour initiating cells Nature 2004 432 396 401 10.1038/nature03128 15549107

[B5] Zhou L Wei X Cheng L Tian J Jiang JJ CD133, One of the Markers of Cancer Stem Cells in Hep-2 Cell Line Laryngoscope 2007 117 455 460 10.1097/01.mlg.0000251586.15299.35 17334305

[B6] Al-Hajj M Wicha MS Benito-Hernandez A Morrison SJ Clarke MF Prospective identification of tumorigenic breast cancer cells Proc Natl Acad Sci USA 2003 100 3983 3988 10.1073/pnas.0530291100 12629218 PMC153034

[B7] Schrot RJ Ma JH Greco CM Arias AD Angelastro JM Organotypic distribution of stem cell markers in formalin-fixed brain harboring glioblastoma multiforme J Neurooncol 2007 85 149 157 10.1007/s11060-007-9401-8 17516028

[B8] Ricci-Vitiani L Ricci-Vitiani L Lombardi DG Pilozzi E Biffoni M Todaro M Peschle C De MariaDe R Identification and expansion of human colon-cancer-initiating cells Nature 2007 445 111 115 10.1038/nature05384 17122771

[B9] O'Brien CA Pollett A Gallinger S Dick JE A human colon cancer cell capable of initiating tumour growth in immunodeficient mice Nature 2007 445 106 110 10.1038/nature05372 17122772

[B10] Smith LM Nesterova A Ryan MC Duniho S Jonas M Anderson M Zabinski RF Sutherland MK Gerber HP Orden Van KL Moore PA Ruben SM Carter PJ CD133/prominin-1 is a potential therapeutic target for antibody-drug conjugates in hepatocellular and gastric cancers Br J Cancer 2008 99 100 109 10.1038/sj.bjc.6604437 18542072 PMC2453027

[B11] Horst D Kriegl L Engel J Jung A Kirchner T CD133 and nuclear beta-catenin: The marker combination to detect high risk cases of low stage colorectal cancer Eur J Cancer 2009 45 2034 2040 10.1016/j.ejca.2009.04.004 19403300

[B12] Horst D Kriegl L Engel J Kirchner T Jung A CD133 expression is an independent prognostic marker for low survival in colorectal cancer Br J Cancer 2008 99 1285 1289 10.1038/sj.bjc.6604664 18781171 PMC2570510

[B13] Kojima M Ishii G Atsumi N Fujii S Saito N Ochiai A Immunohistochemical detection of CD133 expression in colorectal cancer: a clinicopathological study Cancer Sci 2008 99 1578 1583 10.1111/j.1349-7006.2008.00849.x 18754869 PMC11159232

[B14] Wang Q Chen ZG Du CZ Wang HW Yan L Gu J Cancer stem cell marker CD133+ tumour cells and clinical outcome in rectal cancer Histopathology 2009 55 284 293 10.1111/j.1365-2559.2009.03378.x 19723143

[B15] Horst D Kriegl L Engel J Kirchner T Jung A Prognostic significance of the cancer stem cell markers CD133, CD44, and CD166 in colorectal cancer Cancer Invest 2009 27 844 850 10.1080/07357900902744502 19626493

[B16] Li CY Li BX Liang Y Peng RQ Ding Y Xu DZ Zhang X Pan ZZ Wan DS Zeng YX Zhu XF Zhang XS Higher percentage of CD133+ cells is associated with poor prognosis in colon carcinoma patients with stage IIIB J Transl Med 2009 7 56 10.1186/1479-5876-7-56 19583834 PMC2715381

[B17] Choi D Lee HW Hur KY Kim JJ Park GS Jang SH Song YS Jang KS Paik SS Cancer stem cell markers CD133 and CD24 correlate with invasiveness and differentiation in colorectal adenocarcinoma World J Gastroenterol 2009 15 2258 2264 10.3748/wjg.15.2258 19437567 PMC2682242

[B18] Horst D Scheel SK Liebmann S Neumann J Maatz S Kirchner T Jung A The cancer stem cell marker CD133 has high prognostic impact but unknown functional relevance for the metastasis of human colon cancer J Pathol 2009 219 427 434 10.1002/path.2597 19621338

[B19] Cheng JX Liu BL Zhang X How powerful is CD133 as a cancer stem cell marker in brain tumors? Cancer Treat Rev 2009 35 403 408 10.1016/j.ctrv.2009.03.002 19369008

[B20] Zhang M Song T Yang L Chen R Wu L Yang Z Fang J Nestin and CD133: valuable stem cell-specific markers for determining clinical outcome of glioma patients J Exp Clin Cancer Res 2008 27 85 10.1186/1756-9966-27-85 19108713 PMC2633002

[B21] Zeppernick F Ahmadi R Campos B Dictus C Helmke BM Becker N Lichter P Unterberg A Radlwimmer B Herold-Mende CC Stem cell marker CD133 affects clinical outcome in glioma patients Clin Cancer Res 2008 14 123 129 10.1158/1078-0432.CCR-07-0932 18172261

[B22] Maeda S Shinchi H Kurahara H Mataki Y Maemura K Sato M Natsugoe S Aikou T Takao S CD133 expression is correlated with lymph node metastasis and vascular endothelial growth factor-C expression in pancreatic cancer Br J Cancer 2008 98 1389 1397 10.1038/sj.bjc.6604307 18349830 PMC2361715

[B23] Ferrandina G Martinelli E Petrillo M Prisco MG Zannoni G Sioletic S Scambia G CD133 antigen expression in ovarian cancer BMC Cancer 2009 9 221 10.1186/1471-2407-9-221 19583859 PMC3224735

[B24] Song W Li H Tao K Li R Song Z Zhao Q Zhang F Dou K Expression and clinical significance of the stem cell marker CD133 in hepatocellular carcinoma Int J Clin Pract 2008 62 1212 1218 10.1111/j.1742-1241.2008.01777.x 18479363

[B25] Salnikov AV Kusumawidjaja G Rausch V Bruns H Gross W Khamidjanov A Ryschich E Gebhard MM Moldenhauer G Büchler MW Schemmer P Herr I Cancer stem cell marker expression in hepatocellular carcinoma and liver metastases is not sufficient as single prognostic parameter Cancer Lett 2009 275 185 193 10.1016/j.canlet.2008.10.015 19026485

[B26] Salnikov AV Gladkich J Moldenhauer G Volm M Mattern J Herr I CD133 is indicative for a resistance phenotype but does not represent a prognostic marker for survival of non-small cell lung cancer patients Int J Cancer 2010 126 950 958 19676044 10.1002/ijc.24822

[B27] Hao XP Willis JE Pretlow TG Rao JS MacLennan GT Talbot IC Pretlow TP Loss of fragile histidine triad epression in colorectal carcinomas and premalignant lesions Cancer Res 2000 60 18 21 10646844

[B28] Al-Hajj M Cancer stem cells and oncology therapeutics Curr Opin Oncol 2007 19 61 64 17133114 10.1097/CCO.0b013e328011a8d6

[B29] Ferrandina G Petrillo M Bonanno G Scambia G Targeting CD133 antigen in cancer Expert Opin Ther Targets 2009 13 823 837 10.1517/14728220903005616 19530986

[B30] Dirks PB CANCER: Stem cells and brain tumours Nature 2006 444 687 688 10.1038/444687a 17151644

[B31] Setoguchi T Taga T Kondo T Cancer stem cells persist in many cancer cell lines Cell Cycle 2004 3 414 415 15004528 10.4161/cc.3.4.799

[B32] Reya T Morrison SJ Clarke MF Weissman IL Stem cells, cancer, and cancer stem cells Nature 2001 414 105 111 10.1038/35102167 11689955

[B33] Shmelkov SV Butler JM Hooper AT Hormigo A Kushner J Milde T St Clair R Baljevic M White I Jin DK Chadburn A Murphy AJ Valenzuela DM Gale NW Thurston G Yancopoulos GD D'Angelica M Kemeny N Lyden D Rafii S CD133 expression is not restricted to stem cells, and both CD133+ and CD133- metastatic colon cancer cells initiate tumors J Clin Invest 2008 118 2111 2120 18497886 10.1172/JCI34401 PMC2391278

[B34] LaBarge MA Bissell MJ Is CD133 a marker of metastatic colon cancer stem cells? J Clin Invest 2008 118 2021 2024 18497883 10.1172/JCI36046 PMC2391070

[B35] Ieta K Tanaka F Haraguchi N Kita Y Sakashita H Mimori K Matsumoto T Inoue H Kuwano H Mori M Biological and genetic characteristics of tumor-initiating cells in colon cancer Ann Surg Oncol 2008 15 638 648 10.1245/s10434-007-9605-3 17932721

[B36] Vermeulen L Todaro M de Sousa Mello F Sprick MR Kemper K Alea Perez M Richel DJ Stassi G Medema JP Single-cell cloning of colon cancer stem cells reveals a multi-lineage differentiation capacity Proc Natl Acad Sci USA 2008 105 13427 13432 10.1073/pnas.0805706105 18765800 PMC2533206

[B37] Czyzewska J Guzinska-Ustymowicz K Lebelt A Zalewski B Kemona A Evaluation of proliferating markers Ki-67, PCNA in gastric cancers Rocz Akad Med Bialymst 2004 9 Suppl 1 64 66 15638377

